# How Much Can the USA Reduce Health Care Costs by Reducing Smoking?

**DOI:** 10.1371/journal.pmed.1002021

**Published:** 2016-05-10

**Authors:** Wayne Hall, Chris Doran

**Affiliations:** 1 University of Queensland Centre for Youth Substance Abuse Research, Royal Brisbane and Women’s Hospital, Herston, Queensland, Australia; 2 National Addiction Centre, King’s College London, London, United Kingdom; 3 School of Human Health and Social Sciences, Central Queensland University, Brisbane, Queensland, Australia

## Abstract

In this Perspective, Wayne Hall and Chris Doran discuss Lightwood and Glantz’s findings and the implications for tobacco control programs in the US, which are currently poorly funded.

Cigarette smoking causes a wide variety of preventable diseases [1]. Its prevalence has declined substantially since the first US Surgeon General’s report (from 43% in 1965 to 18% today), but it remains a leading cause of preventable death in the United States, where it is responsible for more than 480,000 deaths per year, including nearly 42,000 deaths from secondhand smoke exposure.

Declines in the prevalence of smoking among US adults (18 years of age and older) have slowed in recent years, and very large disparities in tobacco use remain across groups defined by race, ethnicity, educational level, and socioeconomic status and across regions of the country. Moreover, thousands of young people start smoking cigarettes every day, and estimates predict that if smoking continues at the current rate among US youth, 5.6 million of today’s Americans younger than 18 years of age are expected to die prematurely from a smoking-related illness [1].

The total economic cost of smoking in the US is estimated at more than $300 billion a year. This includes nearly $170 billion in direct medical care for adults and more than $156 billion in lost productivity due to premature death and exposure to secondhand smoke [2]. To offset some of this cost, state governments collect $25.8 billion each year from tobacco taxes and legal settlements [1]. How much can these costs be reduced by reducing smoking prevalence?

In the current issue of *PLOS Medicine*, Light and Glantz quantify the extent to which lower rates of smoking in the US might translate into lower health care costs. They have estimated how much, on average, a 1% reduction in smoking prevalence in a US state was associated with reduced health costs in that state a year later. Light and Glantz used a regression analytic approach that took into account correlations between the time series and the effects of other differences between states that may influence state health care expenditure, e.g., population age structure, education, ethnic composition, and the prevalence of risky behavior (e.g., obesity, heavy alcohol use). They have also conducted sensitivity analyses to assess the effects on their results of unmeasured variables (e.g., black market tobacco), different ways of measuring smoking and different ways of statistically modelling the effects.

Their results suggest that a 10% relative reduction in smoking prevalence between a state and the national average in one year was followed by an average $6.3 billion reduction (in 2012 dollars) in health care expenditure the following year. Consistent with this finding, the states with the most rigorous tobacco control policies had a much lower smoking prevalence and lower health care expenditures than states that did not have these policies.

Like all modeling studies, this one has its limitations, as acknowledged by the authors. First, their analysis used aggregate state data on tobacco smoking prevalence and health care costs. We cannot use their results to infer the savings in treatment for individual smokers who become nonsmokers. Second, their analysis probably underestimates the potential health savings from reducing smoking by focusing on the short term savings over 1 to 2 y. This gives greatest weight to cardiovascular and those respiratory diseases with the most rapid reduction in risk after quitting smoking. Their analysis does not take account of the reductions in the risk of disease like lung and other cancers. As the authors note, it is much harder to estimate these longer term savings from reducing smoking prevalence, because these effects will also change the age structure of the population. Conversely, over the very long term, some analyses suggest that reducing smoking prevalence may increase health care costs because it allows more people to survive into old age [3]. Third, a focus on reductions in health care costs also underestimates the full economic savings from reducing smoking, such as improved productivity in former smokers of working age. This may balance the long-term health care costs of those who survive to old age. Fourth, the analysis uses US data and US estimates of specific health care costs. These apply to the unique health care system of a large, wealthy, and technologically advanced society. The results cannot therefore be straightforwardly used as estimates of the economic savings that may be achieved in countries with different economies, populations, and health care systems. Similar studies need to be done in countries with different health care systems and a much higher prevalence of cigarette smoking.

Notwithstanding these limitations, the study shows that reducing population smoking prevalence and the number of cigarettes smoked per day are expected to substantially reduce health care costs over the next year. This makes population-based tobacco control policies a very good form of health care and societal investment by governments. These policies have contributed to reducing the smoking prevalence among adults in countries like Australia from 31% in 1986 [4] to 13% in 2013 [5]. These policies are low cost and easy to implement [6]. Increasing taxes reduces smoking and raises government revenue; smoke free policies are widely supported by the public in many countries and not expensive to enforce. The same is true for restrictions on tobacco industry promotion of cigarettes.

The challenge for tobacco control advocates has been to persuade governments to enact these policies in the face of tobacco industry lobbying, legal challenges, and campaigns to manufacture doubt about the health risks of smoking and the need for tobacco control policies [7,8]. Tobacco control interventions continue to be under-utilized and under-funded in the US. The US$468 million allocated by the states amounts to a small fraction of the $3.3 billion the CDC recommends for all states combined [9]. It would take less than 13% of total state tobacco revenue to meet the CDC recommendations in every state. States that have implemented well-funded, sustained tobacco prevention programs continue to report significant progress, adding to the evidence that these programs work. Florida, with one of the longest running programs, recently reported reducing its high school smoking rate to 6.9% in 2015, one of the lowest ever reported by any US state [10]. Appropriate state expenditure would accelerate the decline in tobacco use in youth and adults and bring forward an end to the tobacco smoking epidemic while saving billions of dollars in avoidable health care costs [9,11].
