# Correction: How Much Can the USA Reduce Health Care Costs by Reducing Smoking?

**DOI:** 10.1371/journal.pmed.1002295

**Published:** 2017-04-12

**Authors:** The PLOS Medicine Staff

It was reported incorrectly that there was an average of $6.3 billion reduction (in 2012 dollars) in health care expenditure the following year. The correct average is $63 billion. The publisher apologizes for the error.

## References

[1] Hall W, Doran C (2016) How Much Can the USA Reduce Health Care Costs by Reducing Smoking? PLOS Medicine 13(5): e1002021 doi: 10.1371/journal.pmed.1002021 27164007 10.1371/journal.pmed.1002021 PMC4862676

